# Alterations in Tear Proteomes of Adults with Pre-Diabetes and Type 2 Diabetes Mellitus but Without Diabetic Retinopathy

**DOI:** 10.3390/proteomes13030029

**Published:** 2025-07-01

**Authors:** Guoting Qin, Cecilia Chao, Shara Duong, Jennyffer Smith, Hong Lin, Wendy W. Harrison, Chengzhi Cai

**Affiliations:** 1 College of Optometry, University of Houston, Houston, TX 77204, USA; 2 Mass Spectrometry Laboratory, Department of Chemistry, University of Houston, Houston, TX 77204, USA; 3 School of Optometry and Vision Science, University of New South Wales, Sydney, NSW 2052, Australia; 4 Department of Mathematics, University of Houston, Houston, TX 77204, USA; 5 Department of Computer Science & Engineering Technology, University of Houston–Downtown, Houston, TX 77002, USA

**Keywords:** tear film, type 2 diabetes mellitus, mass spectrometry, proteomics, machine learning

## Abstract

Background: Type 2 diabetes mellitus (T2DM) is an epidemic chronic disease that affects millions of people worldwide. This study aims to explore the impact of T2DM on the tear proteome, specifically investigating whether alterations occur before the development of diabetic retinopathy. Methods: Flush tear samples were collected from healthy subjects and subjects with preDM and T2DM. Tear proteins were processed and analyzed by mass spectrometry-based shotgun proteomics using a data-independent acquisition parallel acquisition serial fragmentation (diaPASEF) approach. Machine learning algorithms, including random forest, lasso regression, and support vector machine, and statistical tools were used to identify potential biomarkers. Results: Machine learning models identified 17 proteins with high importance in classification. Among these, five proteins (cystatin-S, S100-A11, submaxillary gland androgen-regulated protein 3B, immunoglobulin lambda variable 3–25, and lambda constant 3) exhibited differential abundance across these three groups. No correlations were identified between proteins and clinical assessments of the ocular surface. Notably, the 17 important proteins showed superior prediction accuracy in distinguishing all three groups (healthy, preDM, and T2DM) compared to the five proteins that were statistically significant. Conclusions: Alterations in the tear proteome profile were observed in adults with preDM and T2DM before the clinical diagnosis of ocular abnormality, including retinopathy.

## 1. Introduction

Type 2 diabetes mellitus (T2DM) is an epidemic chronic disease that affects millions of people worldwide [1]. Its ocular manifestations include diabetic retinopathy and macular edema, which are the leading causes of vision loss in working-aged Americans. More recently, the anterior segment changes, including increased corneal thickness, reduced corneal sub-basal nerve fiber density, decreased conjunctival goblet cell density, and increased meibomian gland dropout, have been reported, and are likely associated with diabetic peripheral neuropathy [2,3]. These changes are often associated with ocular discomfort or pain, vision fluctuations, and even serious consequences, including infections and vision loss, thus greatly diminishing patients’ quality of life [4,5]. The current standard of care for ocular complications of diabetes is often palliative and mainly focuses on symptom management, and lacks effective treatment options to reverse vision loss. Consequently, the identification of ocular biomarkers for the early detection of the ocular changes due to diabetes becomes crucial for timely intervention to prevent its progression into severe complications such as retinopathy.

Omics technologies have significantly advanced our understanding of diabetic retinopathy [6,7,8,9]. Among them, metabolomics and lipidomics have revealed key insights into the metabolic dysregulation associated with diabetic retinopathy [10,11]. Proteomics has identified several inflammation mediators in subjects with diabetic retinopathy [12,13,14]. However, many of the early studies often require invasive sampling of ocular fluids such as vitreous humor [15,16,17]. Tear proteomics is a promising tool for population screening as well as biomarker discovery due to its non-invasive collection nature [8,18,19,20,21]. Changes in the tear proteome have been reported under various ocular physiological and pathological conditions such as aging, dry eye, and glaucoma [22,23,24,25,26,27,28]. Moreover, alterations in retinal physiology and pathology have also been shown to induce changes in the protein composition of the tear film [29,30,31,32]. However, it is unclear whether the tear proteome is altered in T2DM patients prior to the occurrence of clinically visible ocular complications. Here, in this work, mass spectrometry-based shotgun proteomics was used to profile tear proteins in healthy, preDM, and T2DM adults. Machine learning models, including random forest, lasso regression, and support vector machine algorithms, were then applied to identify a panel of important features (proteins) that can be potentially used for monitoring ocular manifestations of T2DM.

## 2. Materials and Methods

### 2.1. Materials and Reagents

LC-MS-grade water, acetonitrile, formic acid (FA), and sequencing-grade trypsin were purchased from Thermo Fisher Scientific (Pittsburgh, PA, USA). All other chemicals were purchased from Millipore Sigma (St. Louis, MO, USA) and used without further purification unless noted otherwise.

### 2.2. Subject Recruitment

This was a cross-sectional, single visit study conducted at the University of Houston, College of Optometry. The designed purpose of the study was to evaluate structure and function changes in the anterior and posterior segment in diabetes and prediabetes. Early posterior functional findings are published elsewhere [33]. Tears were gathered as part of an evaluation of the anterior segment across glucose dysfunction levels. Proteomics was not initially planned for the study but was included when additional tears were available for analysis. This study followed the tenets of the Declaration of Helsinki, and the protocol was approved by the Institutional Review Board at the University of Houston prior to study recruitment. Written informed consent was obtained from all subjects prior to their participation.

All subjects were between 30 and 70 years old (50 ± 10 years, data expressed as mean ± standard deviation). The subjects of all races and both sexes were eligible for inclusion. The exclusion criteria for this study included type 1 diabetes, autoimmune diseases, prior eye surgery that may affect the corneal structure, active eye diseases (such as glaucoma, cataract, or macular degeneration), as well as pregnancy.

Subjects were grouped by the standards of the American Diabetes Association, as healthy (<5.7%), preDM (5.7–6.4%), and T2DM (>6.4%) based on their hemoglobin A1c (HbA1c) level measured at the study visit using the Siemens HbA1c analyzer (Siemens, Munich, Germany), as previously described [3]. Contact lens wear was also recorded. All subjects had a monocular corrected distance vision of 20/30 or better in both eyes.

### 2.3. Tear Collection

Twenty microliters of sterile saline was flushed from the temporal canthus of the left eyes. Subjects were then asked to roll their eye clockwise with the eye opened, and the tears were collected from the temporal lower tear meniscus using a clean disposable glass capillary tube (Blaubrand intraMARK, Werthein, Germany). This procedure was repeated two more times on the same eye and the total amount of the flush tears was recorded. Tears were then stored at −80 °C freezer within 30 min until analysis.

### 2.4. Clinical Assessments

The severity of ocular symptoms was determined using the Ocular Surface Disease Index (OSDI) survey [34]. The total score was calculated, and a higher score indicates greater symptoms. The health of the ocular surface, including lids/lashes, conjunctiva, cornea, iris, and lens, was examined via a slit lamp exam, and the findings (normal or abnormal) were recorded for both eyes. The following ocular clinical assessments were only conducted on the left eye. The total central and peripheral corneal thickness was obtained through an Optovue Avanti anterior segment Ocular Coherence Tomographer (OCT) (Optovue, Fremont, CA, USA). The right eye was dilated with tropicamide and phenylephrine for posterior segment testing. The presence or absence of diabetic retinopathy and edema was recorded via a retinal fundus evaluation, which included both a fundus camera (Topcon, Toyko, Japan) and an OCT/OCT-A (Heidelberg Franklin MA) completed on both eyes.

### 2.5. Proteomic Sample Preparation

Tear samples were added with equal volumes of 100 mM ammonium bicarbonate and heated at 95 °C for 5 min for protein denaturation. Proteins were reduced with 5 mM dithiothreitol at 37 °C for 1 h and subsequently alkylated with 10 mM iodoacetamide for 30 min in the dark. Protein concentrations were determined using the Bradford assay, and 2 µg of protein was subjected to tryptic digestion. Trypsin was added at a ratio of 1:40 (enzyme-to-substrate, *w*/*w*; 50 ng) and incubated overnight at 37 °C. The digestion reaction was quenched with trifluoroacetic acid. The resulting peptides were purified using C18 Ziptips and vacuum dried using a CentriVap concentrator (Labconco Corporation, Kansas City, MO, USA).

### 2.6. NanoLC-MS/MS

The liquid chromatography–mass spectrometry (LC-MS) procedure has been described previously [35]. Briefly, analysis was performed using a NanoElute LC system (Bruker Daltonics, Bremen, Germany) coupled with a timsTOF Pro mass spectrometer via a CaptiveSpray ionization source. Peptide samples (200 ng/μL) in 0.1% formic acid were loaded onto an in-house packed analytical column (75 μm × 15 cm, 1.9 μm ReproSil-Pur C18 particle (Dr. Maisch GmbH, Ammerbuch-Entringen, Germany)) and maintained at 40 °C. Mobile phases consisted of buffer A (0.1% formic acid in water) and buffer B (0.1% formic acid in acetonitrile). Peptides were separated using a 21 min gradient: 2% to 30% buffer B over 17.8 min, ramped to 95% B by 18.3 min, and held for an additional 2.4 min. Each sample was analyzed once without additional technical replicates. Data acquisition was performed in the diaPASEF mode [36,37], using 24 *m*/*z* isolation windows per cycle. Electrospray ionization was carried out at 1.6 kV, with the ion transfer tube maintained at 180 °C. Full MS scans were acquired over the mass-to-charge (*m*/*z*) range of 150–1700.

### 2.7. Data Processing

Software Spectronaut v15 (Biosynosis, Zurich, Switzerland) and an in-house human tear spectral library were used with default settings. The in-house human tear spectral library was generated using the previously published procedure [38], with modifications. Tear samples from two projects were utilized to expand the proteome coverage. Specifically, pooled tear samples from each project were fractionated into 16 fractions using high pH reverse-phase fractionation. Data-dependent acquisition (DDA) runs of tear samples were performed using the LC-MS parameters in the above reference [38]. A spectral library was then generated using the built-in library generation function in Spectronaut v15 using default settings. The UniProt SwissProt database (*Homo Sapiens* (Taxon ID 9906), downloaded on 30 January 2021, 42,383 entries) was used. Cysteine carbamidomethylation was used as a fixed modification, and methionine oxidation and acetylation as variable modifications. The FDR was controlled at <1% at peptide spectrum match, peptide, and protein levels. The resulting spectral library contains 14,261 peptides and 3054 proteins.

In the current study, a DIA search was performed using Spectronaut v15. The FDR was also controlled at <1% at peptide spectrum match, peptide, and protein levels. Proteins were considered present if identified by at least two unique peptides that also passed the false discovery rate (FDR) filtering. Protein quantification was performed using the top 3 peptides, and peptide quantification was based on the area of MS2 signals.

### 2.8. Data Analysis

Subject demographic and clinical findings of the continuous data type were tested for normality and compared using one-way ANOVA or the Kruskal–Wallis test based on the normality test. Post hoc analyses were performed using Tukey or Dunn’s test for parametric and non-parametric data. Categorical data were compared using the Chi-square test. Log2-transformation and loess normalization were used to normalize the protein quantification data. All machine learning models were performed using the scikit-learn library (v1.0.2) in Python 3.8. Each model was described below. The hyper parameters were estimated using grid-search, tenfold cross-validation, or the methods described below. The best parameter combination in the model was selected.

Random forest (RF) models for classification and regression were used [39]. Optimization was performed with a depth range of 1–10 and an n_estimators range of 10–300 using the cross-validated grid-search. The optimized values of depth of 2 and n_estimators of 180 were used. Both models were trained on diabetic status with identified protein features. Only proteins with Gini scores above the 98th and 96th percentile were used as important features.

A support vector machine [40] (SVM) model tuned with a linear kernel and epsilon = 0.2 was trained on the protein set to fit the target group labels. Coefficients were assigned to each protein feature, and for linear SVM, higher weights indicate more influence. Proteins with weights in the 96th percentile were selected as important features.

The percentile thresholds for the above models were optimized by evaluating a range of quartile thresholds for 0.90 and 0.99, with 0.01 increments. Proteins identified from all percentile threshold combinations were used for prediction, as described below. The set of percentile thresholds that yielded the highest prediction accuracy is presented here.

Lasso regression penalizes less influential features through L1 regularization [41,42], zeroing out the least important proteins. For scikit-learn’s Lasso function, the alpha parameter that sets the strength of the penalty was optimized within the range of 0.1–1 and set to be 0.14 after optimization. Proteins with non-zero coefficients were used as important proteins.

The important proteins identified in each model were combined. Linear Discriminant Analysis (LDA) was used to confirm the linear separability of the chosen proteins. To evaluate the prediction power of identified proteins, 10-fold cross validation was used and generated an average accuracy. Each sample was used exactly once as the testing data. Protein classification and pathway analysis were performed on identified proteins using the PANTHER (protein analysis through evolutionary relationship) classification system [43,44]. Protein-level abundance comparison analysis was performed using empirical Bayes moderated tests, as implemented in the R/Bioconductor limma package [45], and adjusted for age by incorporating it as a covriate. By including age in the model, limma accounts for the variability in expression that may be due to age, thereby isolating the true effect of the group variable. This adjustment helps to reduce confounding, ensuring that any detected differences between groups are not simply due to the differences in age distribution across the groups. Specifically, the Limma method takes a linear model approach to analyze protein abundance on the covariates. The *p*-values of the moderated t-test of multiple proteins are further adjusted using the Benjamini and Hochberg method. The limma statistical method overcomes the difficulty of small sample sizes to achieve large power and has been used for comparative proteomic data analysis [46]. Only proteins with an adjusted *p*-value below the threshold (alpha = 0.05) were considered statistically significant. The correlation analysis between protein abundance and clinical measurements was performed using Spearman’s rank correlation in the rcorr.adjust function in the RcmdrMisc package. The *p* values were adjusted for multiple inference using the Holm’s method [47], and a *p* value of <0.05 was considered significance. The functional analysis of the gene ontology biological process and reactome pathway terms associated with identified proteins was performed using the ClueGO tool [48] (v2.5.9) in Cytoscape [49] (v3.9.1).

## 3. Results

### 3.1. Subject Demographics and Clinical Characteristics

The demographics and clinical assessments of the healthy, preDM, and T2DM subjects are shown in Table 1. There were no statistical differences in sex and self-reported contact lens wear between groups. There was a significant difference in age between groups (*p* = 0.02, Table 1), with the healthy controls being significantly younger than the preDM (post hoc: *p* = 0.01) and T2DM (post hoc: *p* = 0.02) subjects. There was no significant age difference between the preDM and T2DM subjects. Furthermore, there was no difference in the total score of the OSDI between groups. None of the subjects had any retinopathy signs, including macular edema, or reported having corneal neuropathy.

### 3.2. Proteomic Data

**Figure 1:**
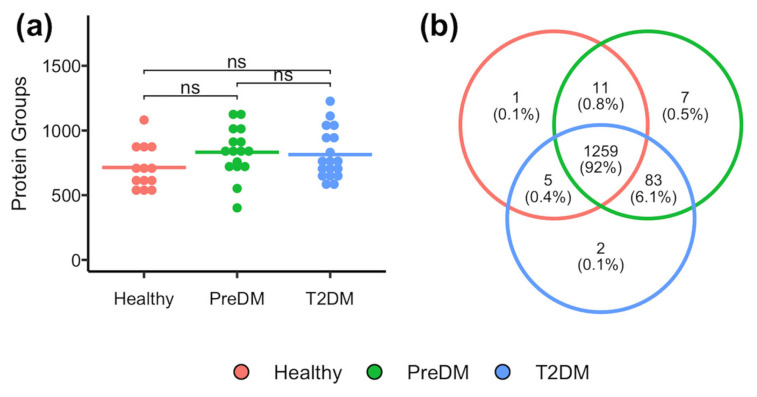
(**a**) The number of identified protein groups in the flush tear of healthy, preDM, and T2DM subjects. “ns” denotes a lack of statistical significance. (**b**) Venn diagram showing the overlap and uniqueness of identified proteins in each subject group.

Using a short 21 min LC gradient, the average number of identified protein groups was 714, 833, and 815 for healthy, preDM, and T2DM subjects, respectively (Figure 1a). There were no significant differences in the number of identified proteins between all three groups. Overall, a total of 1278, 1362, and 1351 protein groups were identified in the tears of healthy, preDM, and T2DM subjects, respectively. Among them, 1261 protein groups were identified in at least one sample from each group, accounting for 92% of the total number of proteins identified in this study (Figure 1b). The high overlapping among the three subject groups indicates that the tear proteome composition was consistent among groups. A complete list of proteins quantified in each sample was included in the Supplementary Materials Table S1.

### 3.3. The Effect of Diabetic Status on Tear Proteome

Machine learning models including random forest (RF) classifier and regression, lasso regression, and support vector machine (SVM) were trained using the 194 protein groups that were quantified across all samples. This relatively low number of proteins included in the analysis was primarily due to the high heteogeneity among subjects. As shown in Figure 1a, the number of proteins identified per sample ranged from 418 to 1403. Because the selection criterion required proteins to be quantified in all samples, the final dataset was constrained by the lowest number of proteins detected in any individual sample. This strigent criterion approach was implemented to enhance the generalizability of the findings across a broader population. Notably, the abundance of these 194 proteins spanned the dynamic range of the tear proteome across all samples (Supplementary Materials Figure S1), ensuring a representative coverage of protein abundance levels.

**Figure 2:**
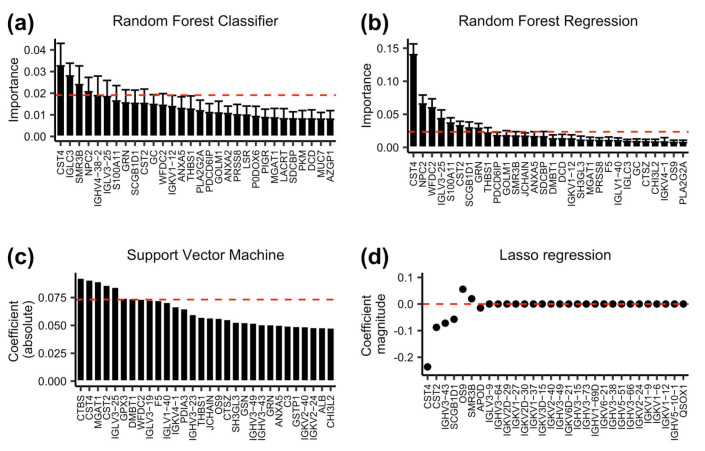
Important proteins identified in each model. Only the top 30 proteins are shown in each model for clean visualization. Feature GINI importance was computed for each protein with (**a**) random forest classifier model, and (**b**) random forest regression model. Data are expressed as Mean ± Standard deviation from 20 simulations. (**c**) Absolute coefficients computed using the support vector machine model with a linear kernel. The dashed line in (**a**–**c**) represented the 98th, 96th, and 96th quartile, respectively. (**d**) Feature coefficients computed using the Lasso regression model.

**Figure 3:**
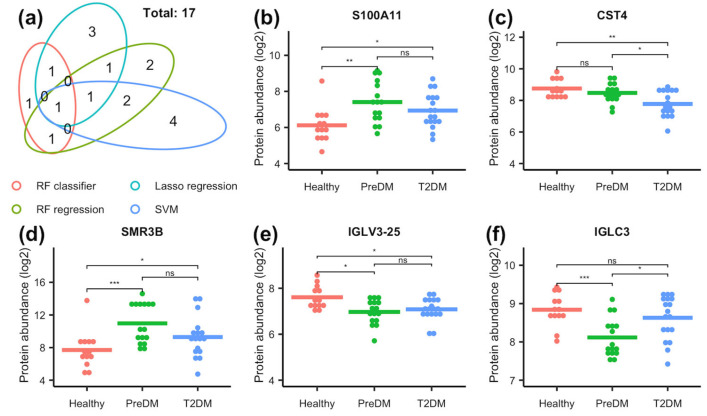
(**a**) Venn diagram showing the uniqueness and overlap of important proteins identified by RF classifier, RF regression, lasso regression, and SVM models. Among the 17 important proteins, protein P31949 (S100A11), P01036 (CST4), P02814 (SMR3B), P01717 (IGLV3-25), and P0DOY3 (IGLC3) (**b**–**f**) showed significantly differential abundance in the tear film of the healthy, preDM, and T2DM subjects. Statistical significance was denoted using the following symbols: “ns” for non-significant results, “*”, “**”, and “***” for *p*-values of < 0.05, ≤ 0.01, and ≤ 0.001, respectively.

**Figure 4:**
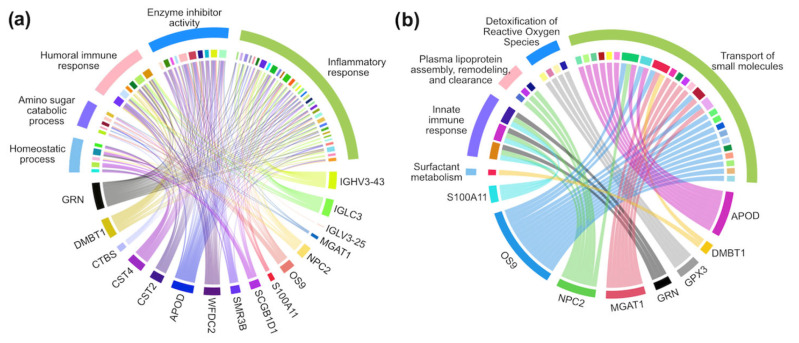
Functional analysis of the 17 important proteins showing their involvement in (**a**) gene ontology biological process (GOBP) and (**b**) reactome pathways. The top of each graph shows GOBP or reactome pathway terms that were grouped for clean visualization. The bottom of each graph shows the proteins involved in each category.

To identify the proteins that play an important role in discriminating the diabetic status in each model, we computed the GINI importance for each protein in the RF models. In the RF classifier model, proteins with a GINI importance score at the top 2% were selected as important proteins, which included CST4, IGLC3, SMR3B, NPC2, IGHV4-38-2, IGLV3-25, S100A11, and GRN (Figure 2a). In the RF regression model, proteins with a GINI importance score at the top 4% were selected as important proteins, which included CST4, NPC2, WFDC2, IGLV3-25, S100A11, CST2, SCGB1D1, and GRN (Figure 2b). For the SVM model, we used the absolute values of coefficient to rank the importance of each protein. The top 4% was determined as important proteins, which were CTBS, CST4, MGAT1, CST2, IGLV3-25, GPX3, A9UGM3, and WFDC2 (Figure 2c). For the Lasso regression model, the important proteins were identified as those with coefficients of non-zero values, which were IGHV3-43, SCGB1D1, CST4, SMR3B, APOD, CST2, and OS9 (Figure 2d). The unique and common proteins identified in each model are shown in Figure 3a, and a total of 17 proteins were identified as important proteins, whose UniProt ID, gene symbol, protein description, and protein class are listed in Table 2. Among the 17 important proteins, protein CST4, IGLV3-25, SMR3B, S100A11, and IGLC3 showed significantly differential abundance in the flush tear of the healthy, preDM, and T2DM subjects (Figure 3b-f). Functional analysis showed their involvement in biological processes such as the inflammatory response, amino sugar catabolic process, and enzyme inhibitor activity (Figure 4a), and reactome pathways such as the transport of small molecules, immune response, removal of reactive oxygen species, and the life cycle of lipoproteins (Figure 4b). Term *p* values are shown in Supplementary Materials Figure S2.

**Figure 5:**
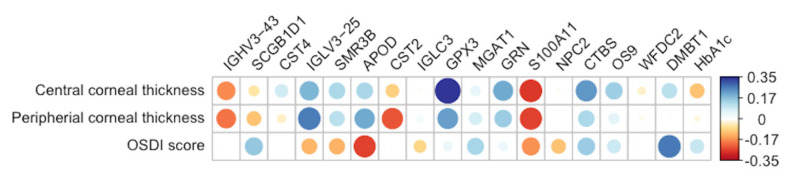
Correlations between protein abundance levels and clinical measurements (central and peripheral corneal thickness and OSDI score). Correlation coefficients are indicated by the color scale bar shown on the right, with positive correlations in shades of blue and negative correlations in shades of red.

Of the 17 important proteins found in this study, the associations with the examined clinical assessments were examined. None of the proteins exhibited significant associations with the central and peripheral corneal thickness and OSDI score (Figure 5).

**Figure 6:**
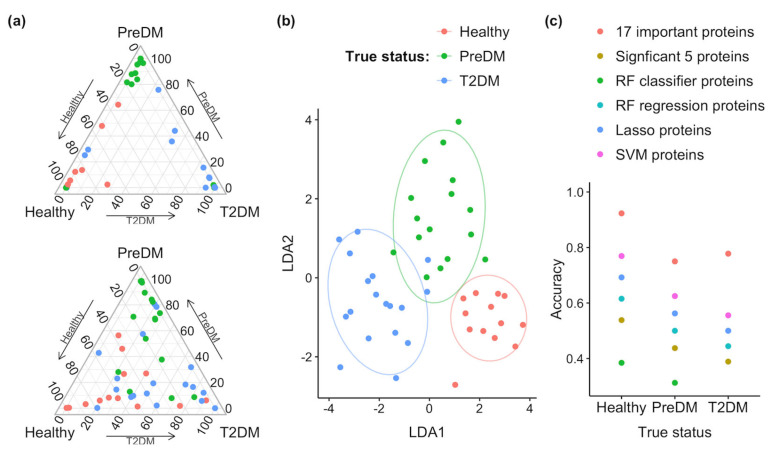
(**a**) Prediction probabilities of each individual sample with its true class in red (Healthy), green (PreDM), and blue (T2DM) using the 17 important proteins (**top**) and 5 statistically significant proteins (**bottom**). (**b**) Discrimination analysis using the 17 important proteins. (**c**) The prediction accuracy of each individual sample using 17 important proteins, 5 statistically significant proteins, and important proteins identified in the RF classifier, RF regression, SVM, and lasso regression models.

Linear discrimination models were then employed with features consisting of a panel of 17 important proteins, 5 statistically significant proteins, and important proteins identified in each machine learning model, respectively. The prediction probabilities are shown in Figure 6a, using the 17 important proteins as features, which showed higher prediction probabilities among the healthy, preDM, and T2DM groups compared to the 5 statistically significant proteins. Figure 6b showed good discrimination of the three groups using the panel of 17 proteins. The accuracy of predicting the diabetic status using the 17-protein panel was 0.92, 0.75, and 0.78, respectively, for samples from the healthy, preDM, and T2DM subjects. In comparison, using the 5-protein panel, the prediction accuracy was only 0.54, 0.44, and 0.39, respectively (Figure 6c). Overall, the feature panel consisting of the 17 important proteins performed better in discriminating the healthy, preDM, and T2DM subjects, compared to the feature panel consisting of 5 statistically significant proteins as well as the important proteins identified in each machine learning model. Notably, the presence of 12 non-statistically significant proteins increased the prediction accuracy of diabetic status.

## 4. Discussion

This study aimed to investigate tear proteome changes in preDM and T2DM subjects before the onset of ocular complications, such as retinopathy. The diabetic status of the participants was determined by blood hemoglobin A1c concentration at the time of testing following the ADA guidelines. There were no statistically significant differences in subject sex and contact lens wear, which are factors known to affect the tear proteome. Age was found to be statistically different among the three groups. As age has been shown to affect the tear proteome [35,50], the statistical analysis to identify significant proteins was adjusted for subject age. Moreover, there were also no statistically significant differences in the OSDI score and other clinical assessments such as corneal thickness, indicating that all subjects had a clinically similar ocular surface health status. Furthermore, no sign of retinopathy or edema was observed in any subjects.

Machine learning holds great potential in elucidating complex gene regulatory networks, the prediction of disease phenotypes, as well as precision medicine [51]. As each machine learning model has its advantages and disadvantages, we employed several supervised machine learning models, including a regression model, to identify the proteins that play an important role in discriminating healthy, preDM, and T2DM status. Each model may capture different aspects of the data and identify partially overlapping yet complementary sets of informative features. By combing results from multiple models, we aim to leverage the strengths of diverse algorithms. This integrative approach may help mitigate model-specific biases and enhance the robustness and generalizability of the selected proteins as potential biomarkers.

The RF model was utilized due to its ability to reduce the variance of prediction while retaining a low bias [39]. A lower bias and variance translate to a reduction in the prediction error and also avoid over-fitting the model to the training data [52]. In the RF classifier model, variable importance analysis identified proteins CST4, IGLC3, SMR3B, NPC2, IGHV4-38-2, IGLV3-25, S100A11, and GRN as particularly useful to discriminate samples from individuals of different groups. Similarly, the RF regression model identified NPC2, WFDC2, IGLV3-25, S100A11, CST2, SCGB1D1, and GRN as significant features for classification prediction. SVM has the advantage of minimizing the empirical classification error and maximizing the geometric marge; therefore, it performs well on noisy data [40,53], and has been widely used in classification problems in genomics and proteomics [52]. Here, using a regression kernel, we identified proteins CTBS, CST4, MGAT1, CST2, IGLV3-25, GPX3, DMBT1, and WFDC2 as being of high importance in classifying the different groups. A generalized regression model with L1 regularization (Lasso regression) estimates the regression coefficients through an L1-norm penalized least squares criterion [41,42]. Lasso penalty is an effective device for continuous model selection, especially in problems where the number of predictors far exceeds the number of observations, which is a common problem in genomics and proteomics [52]. Here, the lasso model identified IGHV3-43, SCGB1D1, CST4, SMR3B, APOD, CST2, and OS9 as important features in the classification of diabetic status. As shown in Figure 3a, the proteins identified in each model exhibited limited overlap with only one protein CST4 identified in all four models, and one protein CST2 identified in three models. Ten proteins were exclusively identified in one of the four models. These results suggest the effectiveness of employing multiple machine learning models in identifying key proteins associated with different disease states, and the complementary and unique contributions of each model in identifying informative features.

The prediction power was highest when all the 17 important proteins were utilized (Figure 6c) for classifying all 3 classes: healthy, preDM, and T2DM. Interestingly, the five statistically significant proteins showed much lower prediction accuracies. This result is intriguing as the remaining 12 proteins, despite not showing statistical differences in abundance among different groups, significantly improved the prediction accuracy. This finding suggests that the combination of proteins that did not exhibit different abundance but still played a role in classification might capture subtle variations or interactions that are relevant to the disease status. Therefore, it highlights the importance of considering a broader range of proteins beyond those showing significant differential abundance in order to achieve a more accurate and comprehensive classification of healthy, preDM, and T2DM individuals.

Functional analysis of the 17 proteins revealed their involvement in immune and inflammatory responses, indicating altered immune response in preDM and T2DM subjects, which have been well known. However, this study shows that such changes are also reflected in tears even before the appearance of symptoms and signs of ocular complications such as corneal neuropathy or diabetic retinopathy. Among the proteins that showed statistically significant difference in abundance across different groups, three of them (S100A11, CST4, and SMR3B) were classified as defense/immune proteins and one as a metabolite interconversion enzyme protein by the PANTHER classification (Table 2). Protein S100A11 (UniProt ID P31949) is a proinflammatory protein and involved in cell cycle, ion channel modulation, and keratinocyte differentiation [54]. Serum S100A11 has been identified to be associated with diabetic status [55] and suggested to be a drug target [56]. Although S100A11 was identified in tears of subjects with ocular diseases such as dry eye and meibomian gland disfunction [22], our work showed the abundance levels of S100A11 in tears was altered in preDM and T2DM subjects even before the appearance of symptoms and signs of diabetic retinopathy. CST4 and SMR3B are considered as potential dry eye biomarkers [30,57,58]. However, in this work, the OSDI scores did not show statistical significance among different groups, suggesting the observed difference of CST4 and SMR3B abundance in this study was not attributed to dry eye status but most likely to diabetic status. This finding, along with another study showing altered CST4 levels in the saliva of subjects of diabetes [59], indicates the systemic alterations of CST4 levels in individuals with T2DM. Overall, these results underscore the systemic impact of diabetes as a complex disease that affects various bodily fluids. Consequently, tear film that can be collected non-invasively presents itself as an attractive option for monitoring diabetes-related changes in patients.

Correlation analysis did not identify any proteins with signficant associations with clinical assessments. This finding was not surprising as none of the clinical assessments showed significant differences among the healthy, preDM, and T2DM groups. It is worth noting that central corneal thickness has been reported to increase with T2DM, especially in subjects with a long history of T2DM and ocular complications such as diabetic retinopathy [60,61,62,63], although it was also reported to be unaffected by diabetic status [64]. This discrepancy may be partly due to subject ethnicity (Asian, African, or Caucasian), disease status (duration, the presence/absence of ocular complications, etc.), and sample size. Nevertheless, our study did not observe differences in central corneal thickness among the groups.

Clinical characteristics such as sex, age, contact lens wear, and dry eye status affect the tear proteome [25,27,50,65,66,67]. Although these factors (except age) were not statistically different among the groups in this study, it is desirable to have age- and sex- matched subjects with balanced characteristics, such as contact lens wear within each group. It is worth noting that the number of female subjects was greater than that of male subjects, although the differences in sex distribution among the groups was not statistically significant. As such, potential sex-related bias may be present in our results. A future study will aim for a more balanced sex distribution among participants. Furthermore, the relatively small sample size (47 subjects) and large number of features analyzed in this study may pose a risk of overfitting the machine learning models. Therefore, additional datasets are necessary to validate the applicability of the 17-protein panel for a prediction of diabetic status. Another limitation of this study is the lack of proteoform analysis. Proteome encompasses a complete set of proteins, including diverse proteoforms resulting from genetic variations, alternatively spliced RNA transcripts, post-translational modifications, and other structural or functional variants. While our study provides valuable insights into the potential alterations in the tear protein abundance of subjects who have T2DM but have not developed diabetic retinopathy, it does not capture the changes in the abundance of different proteoforms. As a result, our findings may not fully reflect the proteome alterations in tears in such subjects. A future study aims to explore certain proteoforms, particularly post-translational modifications [68,69,70] and structural variations [71], in tears to fully understand the impact of T2DM on tear proteomes. One technical limitation is the lack of centrifugation immediately after sample collection, a step that is often employed to remove cells or cellular debris from tear samples. Eliminating these particulates may reduce potential confounding factors and enhance the robustness and reproducibility of the results. Nevertheless, we identified five proteins with differential abundance among different groups. Previous studies reported that some of the proteins exhibited altered abundance in the serum or saliva of subjects of diabetes compared to healthy controls, as discussed above. Therefore, our study provides additional support for their relevance to diabetes and for the potential use of tears for monitoring diabetic status.

## 5. Conclusions

In this work, we used supervised machine learning models, including random forest, lasso regression, and support vector machine, and identified a panel of 17 proteins from tears that can be potentially used as biomarkers for the classification of patients with early diabetes mellitus (preDM or T2DM). Five proteins exhibited differential abundance in the tears of the preDM and T2DM groups compared to the healthy controls. Linear discrimination analysis showed that the panel of 17 proteins had higher accuracy in predicting diabetic status compared to the 5 statistically significant proteins. Overall, our results suggest that tear proteome changes occur in preDM and T2DM individuals before the appearance of symptoms and signs of diabetic retinopathy. This finding indicates that T2DM affects the eye before any clinically detectable pathology, emphasizing the early impact of T2DM on ocular physiology. In conclusion, our investigation into the tear proteome of preDM and T2DM individuals, prior to the onset of ocular complications, sheds light on the potential use of tear biomarkers for the early detection and monitoring of diabetes-related ocular changes. Further studies are imperative to validate and expand upon our results, potentially leading to the development of non-invasive diagnostic tools for individuals at risk of diabetic ocular complications.

## Figures and Tables

**Table 1:** Subject demographic data.

	Healthy Control (n = 13)	PreDM (n = 16)	T2DM (n = 18)	*p* Value
Hemoglobin A1c (%)	5.3 (5.1–5.4)	5.8 (5.8–5.9)	7.1 (6.7–7.8)	<0.001
Sex (Female/Male)	9/4	14/2	12/6	0.33
Age (years)	46 (42–52)	59 (50–66)	58 (54–59)	0.02
OSDI score (0–100)	12.5 (2.5–18.8)	7.5 (6.3–15.1)	13.6 (5.5–21.3)	0.57
CLW (Yes/No)	4/9	0/16	4/14	0.06
Central corneal thickness (OS)	533 (523–559)	522 (500–544)	534 (506–568)	0.53
Peripheral corneal thickness (OS)	687 (671–726)	675 (663–699)	704 (671–748)	0.33

Data presented in median and interquartile range (IQR) or frequency counts; OSDI: the Ocular Surface Disease Index; CLW: contact lens wear.

**Table 2:** The 17 important proteins identified from machine learning models with PANTHER protein classification. Proteins with differential abundance among groups are shown in bold font.

UniProt ID	Gene	Description	Protein Class
A0A0B4J1X8	IGHV3-43	Immunoglobulin heavy variable 3-43	calcium-binding protein
O95968	SCGB1D1	Secretoglobin family 1D member 1	defense/immunity protein
P01036	CST4	Cystatin-S	defense/immunity protein
P01717	IGLV3-25	Immunoglobulin lambda variable 3-25	defense/immunity protein
P02814	SMR3B	Submaxillary gland androgen-regulated protein 3B	defense/immunity protein
P05090	APOD	Apolipoprotein D	protein-binding activity modulator
P09228	CST2	Cystatin-SA	protein-binding activity modulator
P0DOY3	IGLC3	Immunoglobulin lambda constant 3	protein-binding activity modulator
P22352	GPX3	Glutathione peroxidase 3	transfer/carrier protein
P26572	MGAT1	Alpha-1,3-mannosyl-glycoprotein 2-beta-N-acetylglucosaminyltransferase	transfer/carrier protein
P28799	GRN	Progranulin	protein-modifying enzyme
P31949	S100A11	Protein S100-A11	metabolite interconversion enzyme
P61916	NPC2	NPC intracellular cholesterol transporter 2	Unclassified
Q01459	CTBS	Di-N-acetylchitobiase	Unclassified
Q13438	OS9	Protein OS-9	Unclassified
Q14508	WFDC2	WAP four-disulfide core domain protein 2	Unclassified
Q9UGM3	DMBT1	Deleted in malignant brain tumors 1 protein	protein-modifying enzyme

## Data Availability

The raw MS data and search results were deposited to the ProteomeXchange Consortium [72] via the PRIDE [73] partner repository with the dataset identifier PXD062366.

## Supplementary Materials

The following supporting information can be downloaded at: https://www.mdpi.com/article/10.3390/proteomes13030029/s1, Figure S1: The distribution of the 194 proteins across the proteome dynamic range of all 47 subjects. The 194 proteins are highlighted in red while all other proteins are shown in gray. Figure S2: The involvement of the 17 important proteins in a) gene ontology biological process (GOBP) and b) reactome pathways. Figure S3: The number of unique peptides of proteins identified within each group. Table S1: A complete list of proteins identified in each sample with abundance. Table S2: The effect of age on the abundance of the 17 important proteins.

## Abbreviations

The following abbreviations are used in this manuscript:

T2DM	Type 2 Diabetes Mellitus
preDM	Pre-type 2 Diabetes Mellitus
diaPASEF	Data-Independent Acquisition Parallel Acquisition Serial Fragmentation
OSDI	Ocular Surface Disease Index
FA	Formic Acid
OCT	Ocular Coherence Tomographer
RF	Random Forest
SVM	Support Vector Machine
