# Supplementary material for: Alterations in Tear Proteomes of Adults with Pre-Diabetes and Type 2 Diabetes Mellitus but Without Diabetic Retinopathy
Source: Proteomes. 2025 Jul 1;13(3):29. doi: 10.3390/proteomes13030029 (PMC12286239; doi:10.3390/proteomes13030029)
Supplement: Supplementary file 1 [file proteomes-13-00029-s001.zip › Supplementary Material.pdf]

Supplementary Material

Alterations in tear proteomes of adults with pre-diabetes and type 2 diabetes mellitus but without diabetic retinopathy

Guoting Qin[1,2,*], Cecilia Chao[1,3] , Shara Duong[4] , Jennyffer Smith[1], Hong Lin[5] , Wendy Harrison[1] , and Chengzhi Cai[2,*]

[1] College of Optometry, University of Houston, Houston, TX 77204

[2] Mass Spectrometry Laboratory, Department of Chemistry, University of Houston, Houston, TX 77204

[3] School of Optometry and Vision Science, University of New South Wales, Sydney, Australia

[4] Department of Mathematics, University of Houston, Houston, TX 77204

[5] Department of Computer Science & Engineering Technology, University of Houston – Downtown, Houston, TX 77002

*Correspondence: GQ, gqin@central.uh.edu. CC, cai@uh.edu.

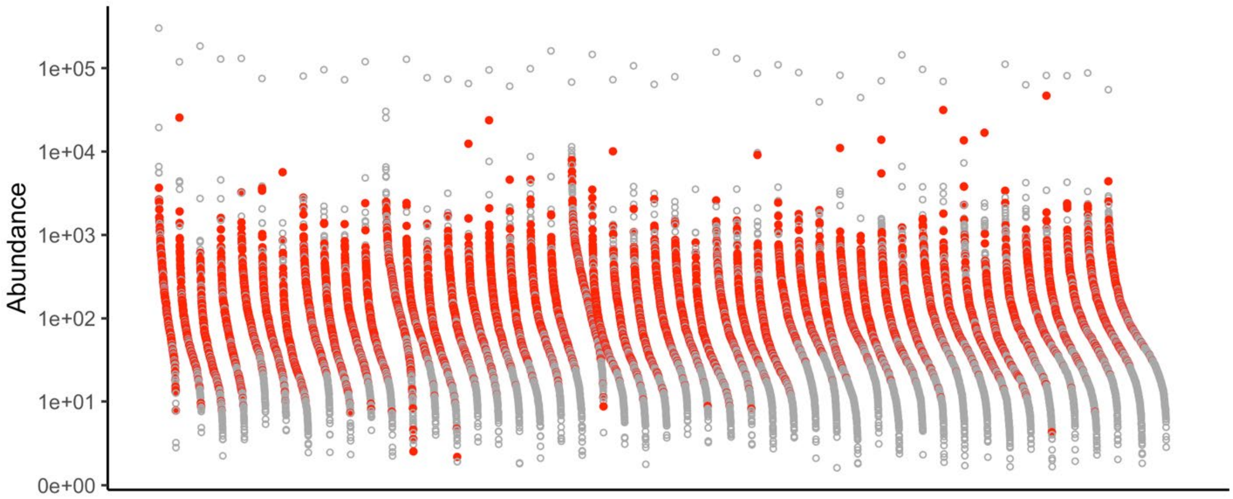

Figure S1. The distribution of the 194 proteins across the proteomic dynamic range of all 47 subjects. The 194 proteins are highlighted in red while all other proteins are shown in gray.

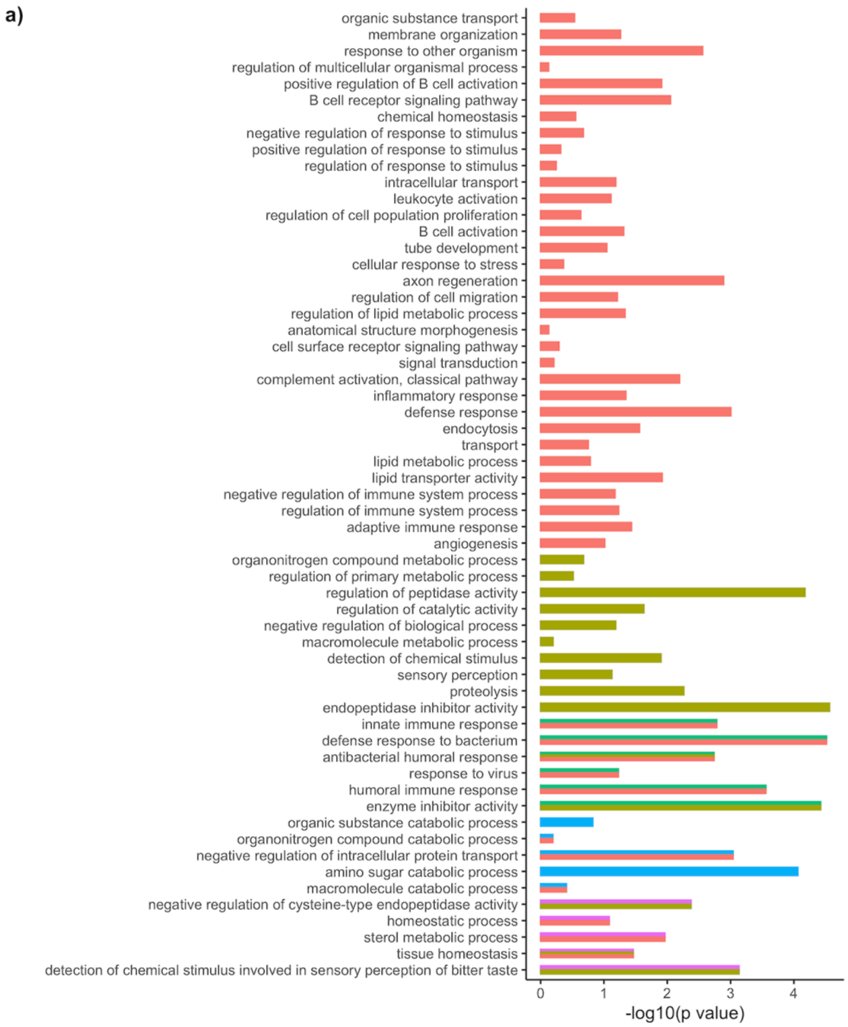

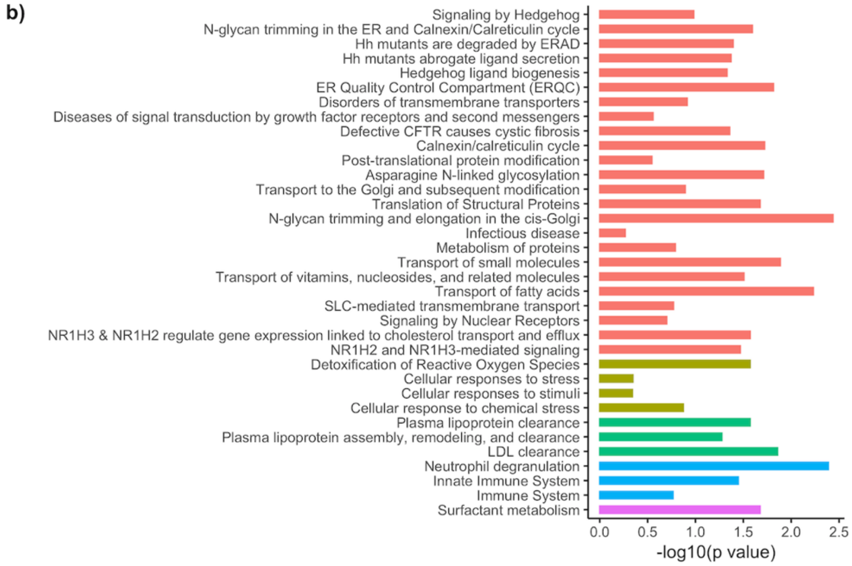

Figure S2. The involvement of the 17 important proteins in a) Gene ontology biological process (GOBP) and b) Reactome pathways.

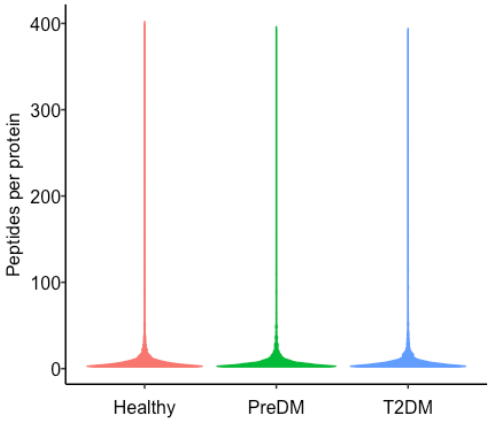

Figure S3. The number of unique peptides of proteins identified within each group.

Table S2. The effect of age on the abundance of the 17 important proteins.

**Gene**	**adj.P.Val**
IGHV3-43	0.89
SCGB1D1	0.90
CST4	0.70
IGLV3-25	0.59
SMR3B	0.59
APOD	0.89
CST2	0.89
IGLC3	0.40
GPX3	0.40
MGAT1	0.89
GRN	0.40
S100A11	0.40
NPC2	0.89
CTBS	0.59
OS9	0.89
WFDC2	0.89
DMBT1	0.89
